# The *GAMYB* gene in rye: sequence, polymorphisms, map location, allele-specific markers, and relationship with α-amylase activity

**DOI:** 10.1186/s12864-020-06991-3

**Published:** 2020-08-24

**Authors:** Anna Bienias, Magdalena Góralska, Piotr Masojć, Paweł Milczarski, Beata Myśków

**Affiliations:** Department of Plant Genetics, Breeding and Biotechnology, West Pomeranian University of Technology in Szczecin, Szczecin; ul, Słowackiego 17, 71-434 Szczecin, Poland

**Keywords:** DArTseq, High-density genetic map, RNAseq, *Secale cereale* L., *ScGAMYB*, SNP, transcription factor

## Abstract

**Background:**

Transcription factor (TF) GAMYB, belonging to MYB family (named after the gene of the avian **my**elo**b**lastosis virus) is a master gibberellin (GA)-induced regulatory protein that is crucial for development and germination of cereal grain and involved in anther formation. It activates many genes including high-molecular-weight glutenin and α-amylase gene families. This study presents the first attempt to characterize the rye gene encoding GAMYB in relation to its sequence, polymorphisms, and phenotypic effects.

**Results:**

*ScGAMYB* was mapped on rye chromosome 3R using high-density Diversity Arrays Technology (DArT)/DArTseq-based maps developed in three mapping populations. The *ScGAMYB* sequences were identified in RNA-seq libraries of four rye inbred lines. The transcriptome used for the search contained almost 151,000 transcripts with a median contig length of 500 nt. The average amount of total base raw data was approximately 9 GB. Comparative analysis of the *ScGAMYB* sequence revealed its high level of homology to wheat and barley orthologues. Single nucleotide polymorphisms (SNPs) detected among rye inbred lines allowed the development of allele specific-PCR (AS-PCR) markers for *ScGAMYB* that might be used to detect this gene in wide genetic stocks of rye and triticale. Segregation of the *ScGAMYB* alleles showed significant relationship with α-amylase activity (AMY).

**Conclusions:**

The research showed the strong similarity of rye *GAMYB* sequence to its orthologues in other *Graminae* and confirmed the position in the genome consistent with the collinearity rule of cereal genomes. Concurrently, the *ScGAMYB* coding sequence (cds) showed stronger variability (24 SNPs) compared to the analogous region of wheat (5 SNPs) and barley (7 SNPs). The moderate regulatory effect of ScGAMYB on AMY was confirmed, therefore, *ScGAMYB* was identified as a candidate gene for partial control of α-amylase production in rye grain. The predicted structural protein change in the aa region 362–372, caused by a single SNP (C/G) at the 1100 position in *ScGAMYB* cds and single aa sequence change (S/C) at the 367 position, is the likely cause of the differences in the effectiveness of ScGAMYB regulatory function associated with AMY. The development of sequence-based, allele-specific (AS) PCR markers could be useful in research and application.

## Background

The expression of structural genes encoding α-amylase and other hydrolases in the aleurone layer of germinating grains is enhanced by a TF GAMYB, through its direct binding to a highly conserved GA-responsive element (GARE, TAACAA/GA) in the promoter [1]. The constitutive expression of *GAMYB* in aleurone cells in the absence of GA is sufficient to activate an α-amylase promoter. Moreover, silencing or loss-of-function mutations of the *GAMYB* suppressed AMY in GA-treated aleurone cells [2, 3]. Thus, GAMYB activity is indispensable for elevated expression of α-amylase genes in response to GA [4]. GAMYB is also involved in production of storage proteins during grain development [5, 6] and in developmental mechanisms of anther formation [4, 7].

GAMYB production in the aleurone layer is controlled by the quantitative ratio of GA and abscisic acid (ABA). Genes encoding GAMYB are suppressed by the ABA signal transduced by protein kinase PKABA1 [8] and by GAMYB-binding protein KGM, which are members of MAK-type protein kinases [9]. GA induces a rapid increase in *HvGAMYB* expression in barley aleurone layers through degradation of its repressor SLN1 belonging to the DELLA proteins [4, 10, 11].

There is only one copy of *GAMYB* per cereal genome, and it is located in the syntenic position on homologous group 3 chromosomes in barley and wheat [12] and on collinear rice chromosome 1 [13]. Sequences of *Hv-* and *TaGAMYB* comprise four exons and three introns that are differentiated to several haplotypes within wide germplasm collections of both species [12].

Functional polymorphisms in *GAMYB* may be an important factor affecting variation of AMY and possibly other important traits of cereals. This possibility should be explored because wheat, barley, and rye chromosome 3 was shown to contain a number of quantitative trait loci (QTLs) for AMY, preharvest sprouting (PHS), and plant height [14–19]. However, the *ScGAMYB* sequence, polymorphisms, map location, and relationship with agronomic traits have not yet been characterized in rye. The aim of this work was to reveal the cds and gene structure of the important TF - GAMYB, and to align the *ScGAMYB* sequence with orthologs identified in wheat and barley. We describe here the location of the studied gene on genetic maps of three mapping populations and its relationship to AMY.

## Results

### Sequence and structure of ScGAMYB examined in rye inbred lines of various origins

The *GAMYB* sequence was established for six rye inbred lines (DS2, RXL10, M12, L35, 541, and Ot1–3) collected from the Department of Plant Genetics, Breeding, and Biotechnology of the West-Pomeranian University of Technology, Szczecin. Four of these lines were used in the entire transcriptome sequencing experiment. RNA was isolated from six plants of the DS2 and M12 and from four plants of the L35 and RXL10. RNA integrity number (RIN) measured before sequencing was appropriate for 17 samples, indicating good RNA quality. RIN of three RNA samples was below 5.5 and these ones were excluded from the experiment.

The average amount of total base raw data was: 9.6 Gb, 9.4 Gb, 8.4 Gb, and 8.1 Gb for line M12, DS2, L35, and RXL10, respectively. Read count ranged from 68,259,118 to 115,869,622 paired end reads. Raw data were subjected to quality control. More than 95% bases were of high quality (Table 1). The raw reads data for 17 samples in FASTQ format were deposited in the National Centre for Biotechnology Information (NCBI) Sequence Read Archive (SRA) database under the accession number from SRX2636904 to SRX2636920. After removing the adapter and low quality sequences from the raw data, from 66.7 to 112.9 million high-quality reads were retained (Table 1).

**Table 1:** Result of data processing of RNAseq performed for four rye inbred lines using Illumina HiSeq 2000 system

Sample	Raw Data			Trimmed Data		
	Total Bases	Read Count	Q30 (%)	Total Bases	Read Count	Q30 (%)
M12w1	11,702,831,822	115,869,622	96.27	11,314,892,610	112,863,536	97.56
M12w2	9,580,521,852	94,856,652	96.61	9,305,022,511	92,771,556	97.71
M12wl1	8,383,791,436	83,007,836	96.39	8,121,465,377	80,988,190	97.61
M12wl2	10,188,945,852	100,880,652	96.52	9,902,654,357	98,700,562	97.64
M12wl3	8,262,443,572	81,806,372	96.44	8,012,781,602	79,886,624	97.63
DS2w1	8,990,270,782	89,012,582	96.7	8,745,071,781	87,164,468	97.75
DS2w2	8,919,988,720	88,316,720	96.46	8,651,277,777	86,255,968	97.63
DS2wl1	9,516,783,378	94,225,578	96.4	9,221,235,694	91,939,090	97.6
DS2wl2	9,282,121,392	91,902,192	96.42	8,997,604,730	89,717,130	97.61
DS2wl3	10,305,669,936	102,036,336	96.01	10,003,200,236	99,819,220	97.19
RXL10w1	9,914,476,130	98,163,130	96.39	9,618,122,251	95,877,590	97.57
RXL10w2	8,362,917,362	82,801,162	95.97	8,111,094,232	80,948,782	97.18
RXL10wl1	6,894,170,918	68,259,118	95.69	6,680,084,234	66,723,890	96.95
RXL10wl2	7,355,772,834	72,829,434	96.44	7,133,202,875	71,110,558	97.61
L35w3	7,553,455,084	74,786,684	96.39	7,319,205,497	72,975,450	97.59
L35wl3	8,013,652,696	79,343,096	96.59	7,795,961,803	77,714,250	97.65
L35wl	9,717,103,344	96,208,944	96.58	9,444,304,014	94,146,958	97.66

The quality of the sequences measured with Q30 ranged from 96.95 to 97.75% (Table 1). The 17 set clean reads of the four genotypes were de novo assembled into one transcriptome with the Trinity program [20]. The constructed reference sequence pool counted 218,935 transcripts with a total nucleotide number of 183.8 million (Table 2).

**Table 2:** De novo transcriptome assembly statistics with Trinity program for 17 RNA samples of four rye inbred lines

Sample	All transcript contigs	Only longest isoform per ‘GENE’
Total trinity ‘genes’	150,758	150,758
Total trinity transcripts	218,935	150,758
N50	1366	1366
Maximum contig length	16,024	16,024
Minimum contig length	201	201
Median contig length	500	401
Average contig length	839.29	703.25
Total assembled bases	183,750,947	106,021,123

The average contig length was 840 nt, with a minimum of 200 and a maximum of 16,024 nt. The statistics N50 was 1366 base pairs, including all transcripts obtained (Table 2). About 150,000 genes with longest isoform was identified (Table 2). Functional annotations performed using GO and eggNOG databases revealed 32,152 transcripts (15%) linked to biological processes, 25,998 (12%) - to cellular components, and 25,281 (11%) - to molecular function. A number of 135,503 transcripts were not assigned any function.

Based on the annotation, several contigs identified as MYB were identified, but it was not known which might correspond to the GAMYB, so it was decided to search the whole database of 218,935 transcripts at the nucleotide sequence level, to reveal a contig containing the GAMYB TF sequence. The cds of the *GAMYB* identified in barley (AY008692.1) was used for this purpose. The alignment performed by the Geneious software version 10.2.4. with megablast tool, resulted in identification of contig No. c81081_g3_i1 containing the *GAMYB* cds (Additional file 1). The annotation assigned to the contig pointed to the following functions: evidence of DNA binding, evidence of chromatin binding, evidence of transcription factor activity binding DNA sequence, nucleus evidence, transcription regulation, DNA template evidence, evidence of gibberellic acid mediated signaling pathway, pollen cell evidence of differentiation positive regulation, ABA activated signaling pathway, positive regulation of evidence for programmed cell death, positive transcription regulation, evidence based on DNA template, negative regulation of growth evidence, evidence of GA biosynthesis.

The length of contig c81081_g3_i1 was 1816 nt and its sequence contained both exon 2, 3 and 4, together with the 5 ‘UTR sequence. As this contig did not cover the entire sequence of the *GAMYB* described in wheat and barley (lack of exon 1), the search of the contig database was repeated, using only the first exon sequence identified in barley (AY008692.1). Based on this activity, contig c81081_g4_i1 (Additional file 1) was identified, containing only the exon 1 sequence. Mapping both contigs to the *GAMYB* sequence of related species and the rye sequence of the Lo7 line [21] allowed to assemble the full mRNA sequence of the *ScGAMYB*.

The alignment of the two rye Sc*GAMYB cDNA* sequences gave a total length of 2241 nt for M12 and DS2 lines. Sequences for RXL10 and L35 were shorter. Based on the raw data of reads, length of exon 1 in RXL10 and L35 lines was determined to be 240 and 54 nt respectively. These four sequences contained the entire cds of *ScGAMYB* – of 1659 nt.

The 769 nt fragment of *ScGAMYB* of lines 541 and Ot1–3 was amplified from genomic DNA, with primers pair F: 5′-tatgcaccacagctttcagc-3′, R: 5′-gtgcaggaggaattttggag-3′, because these lines were not included in the rye transcriptome study. This fragment corresponds to exon 3 of the studied gene. *ScGAMYB* nucleotide sequences showed 99–100% identity with orthologous genes of wheat and barley deposited in the NCBI database (Table 3).

**Table 3:** Similarity of *ScGAMYB* coding sequence (cds) with sequences of related cereal species - NCBI BLAST analyze results

Species	Max Score	Query cover	% Identity	Accession no.
*Hordeum vulgare*	1277	99%	96.74	AY008692.1
*Triticum aestivum*	1279	100%	96.63	JF951917.1
*Triticum monococcum*	1254	100%	96.10	AB214883.1
*Avena sativa*	878	99%	87.50	AJ133638.1
*Brachypodium distachyon*	852	100%	86.68	XM_003564404.3

The resulting *ScGAMYB* mRNA sequence was aligned to the whole genome shotgun sequence assembly of rye inbred line Lo7 [21]. The *Sc170168* was a DNA fragment (scaffold) containing the entire sequence of *ScGAMYB* located on chromosome 3R at position 92.15706326 cM on the rye genetic map constructed previously [21]. The identity coefficients between the six rye inbred lines *GAMYB* sequences and those found within the scaffold were from 96.56 to 97.46%.

**Fig. 1:**
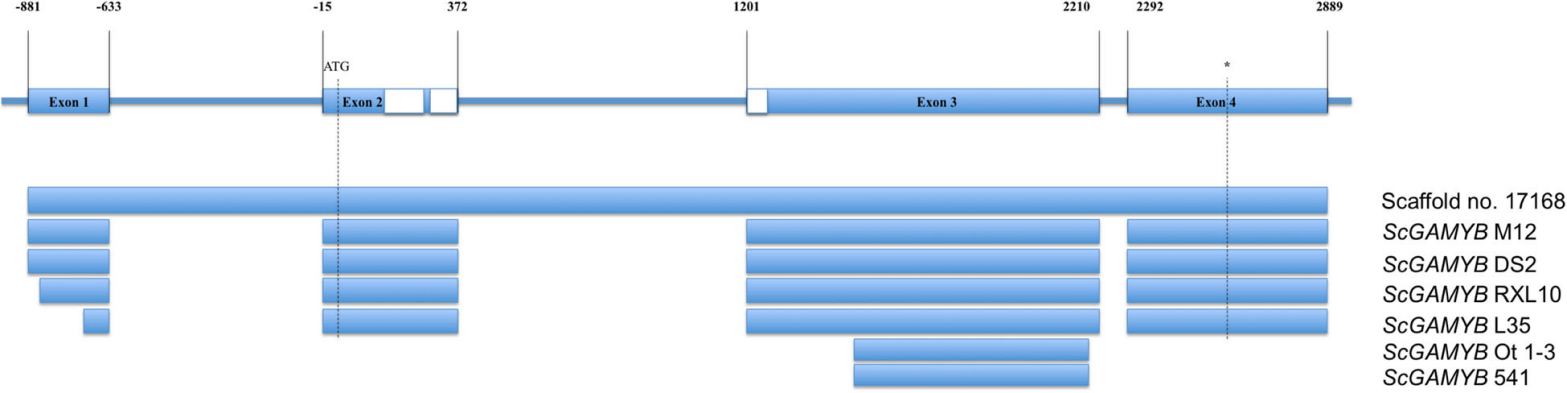
The structure of the *ScGAMYB* gene determined based on the sequence data of six studied rye inbred lines with respect to the genomic sequence of the Lo7 inbred line scaffold [21]

The structure of *ScGAMYB* (Fig. 1) was determined by comparing the sequencing nucleotide data reported in this study with the rye DNA sequences presented by Haseneyer et al. [12]. It has a total length of 3700 nt and contains four exons and three introns similarly to the cds of orthologous genes in wheat and barley. The particular exons of *ScGAMYB* sequence contain: 248 nt, 387 nt, 1009 nt, and 597 nt being interspaced by three introns of 618 nt, 829 nt, and 82 nt lengths with the cds spanning from exon 2 to exon 4 (Fig. 1).

Upon comparing the mRNA sequence of the *ScGAMYB* of the DS2, RXL10, L35, and M12 rye lines, we noted that exon 3 has the highest variability (Additional file 2, Table 4). Comparative analysis of the *ScGAMYB* chromatograms representing a gene fragment of 769 nt length revealed four SNPs differentiating Ot1–3 and 541 lines (C-T, A-G transitions at positions 470 and 476, respectively, and G-C transversions at 485 and 495 positions of the *ScGAMYB* fragment sequence). The identified point mutations (Additional file 2, Table 4) were used to design primers to generate AS products (Table 5). AS-PCR markers uncovered polymorphisms not only between 541 and Ot1–3, but also between two other parental pairs of lines (DS2 and RXL10; S32N/07 and RXL10) and within mapping populations created from crossing these lines. A total of 10 primer pairs were designed (Table 5). All of them allowed one to amplify stable and repeatable products specific to the alleles tested.

**Table 4:** Polymorphisms between rye inbred lines in ScGAMYB sequence detected in nucleotide (nt) sequence, expected amino acid (aa) sequence and predicted protein second-structure (2D)

changed nt position	type of nt change*	line with change	changed aa position	type of aa change	line with change	change in 2D
Lines: DS2, L35, M12, RXL10						
99	C → T	DS2	33	–	–	–
129	G → A	DS2	43	–	–	–
204	C → G	DS2	68	–	–	–
226, 227	AA→TT	M12	76	N → F	M12	+
259–261	ATC → GCG	M12	87	I → A	M12	+
265	T → C	M12	89	Y → H	M12	+
271	C → A	M12	91	–	–	–
277	G → A	M12	93	D → N	M12	–
564	C → T	DS2	188	–	–	–
Lines: 541, Ot1–3, DS2, L35, M12, RXL10						
681	C↔T	L35, M12, RXL10	227	–	–	–
698	A↔C	L35, M12, RXL10	233	Q↔P	L35, M12, RXL10	+
720	A↔G	L35, M12, RXL10	240	–	–	–
735	G↔A	L35, M12, RXL10	245	–	–	–
741	C↔T	L35, M12, RXL10	247	–	–	–
944	G↔A	L35, M12, RXL10	315	S↔N	L35, M12, RXL10	–
**1100**	**C → G**	**541, DS2**	**367**	**S → C**	**541, DS2**	**+**
1110	G → C	541, DS2	370	–	–	+
1185	C → T	541, DS2	395	–	–	–
1190, 1191	GG → GA/AG	541/DS2	397	R → K	DS2	+
1375	T → C	541,Ot1–3	459	S → P	541, Ot1–3	–

*The unidirectional arrows were used when the change concerned a minority of lines from the set of four or six lines tested. Changes important for the 2D protein structure and related to the effect of ScGAMYB on AMY are bolded

**Table 5:** Characteristics of AS-PCR primers for *ScGAMYB* identification in rye, differentiating rye inbred lines used as parental components of three mapping populations: BSR-F_2_ (S32N/07 × RXL10), RIL-K (541 × Ot1–3)), and RIL-L (DS2 × RXL10)

Common primer		AS primer designed to 541 line sequence			AS primer designed to Ot1–3 line sequence			SNP position (cds / exon 3 fragment)	Product size (bp)
Name	Sequence	Name	Sequence	Allele presented in line	Name	Sequence	Allele presented in line		
31F20	catcaggcgacgcagtgctc	541.285R20c	ccgtcagtgaaatcggagtc	541, DS2, S32N/07	Ot.285R20g	ccgtcagtgaaatcggagtg	Ot1–3	1100/485	274
		541.275R20g	aatcggagtstgcgtcgccg	541, DS2	Ot.275R20c	aatcggagtctgcgtcgccc	Ot1–3	1110/495	264
17F21	ggagagctgaaaaacatcagg	541.285R20c	ccgtcagtgaaatcggagtc	541, DS2, S32N/07	Ot.285R20g	ccgtcagtgaaatcggagtg	Ot1–3, RXL10	1100/485	288
		541.275R20g	aatcggagtstgcgtcgccg	541, DS2	Ot.275R20c	aatcggagtctgcgtcgccc	Ot1–3, RXL10	1110/495	278
583R24	cggtcgatcagttctcaaatgact	541.266F20c	gctgttcctcggcgacgcac	541, DS2, S32N/07	Ot.266F20g	gctgttcctcggcgacgcag	Ot1–3, RXL10	1100/485	341

**Fig. 2:**
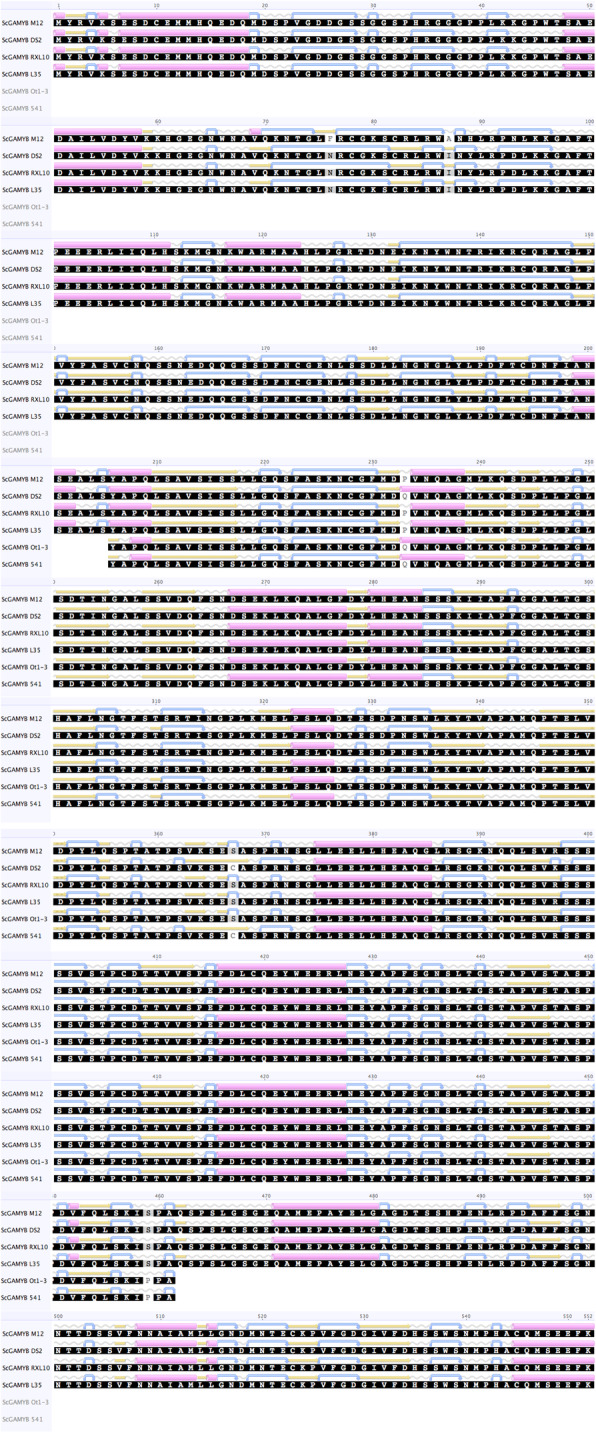
The changes in secondary structure of ScGAMYB of rye inbred lines resulting from SNPs (the protein structure predicted with EMBOSS 6.5.7 plug)

Comparative analysis of the six sequences revealed 24 SNPs in cds of *ScGAMYB* (Additional file 2, Table 4). This 1659 nt cds was translated into a 552 amino acid (aa) protein sequence (Additional file 3, Table 4) using Geneious software. The analysis revealed that some SNPs resulted in a change of the aa sequences at positions 76 (F/N), 87 (A/I), 233 (P/Q), 367 (S/C), and 459 (S/P) (Additional file 3, Table 4). In addition, the protein structure prediction conducted by EMBOSS 6.5.7 plug showed that these polymorphisms affected the secondary structure of *ScGAMYB* (Fig. 2). Moreover, two SNPs resulted in a change of protein sequence in the 44–93 aa region that codes the functional MYB domain and is responsible for DNA-binding. The second region of DNA-binding domain (97–144 aa) was highly conserved.

**Fig. 3:**
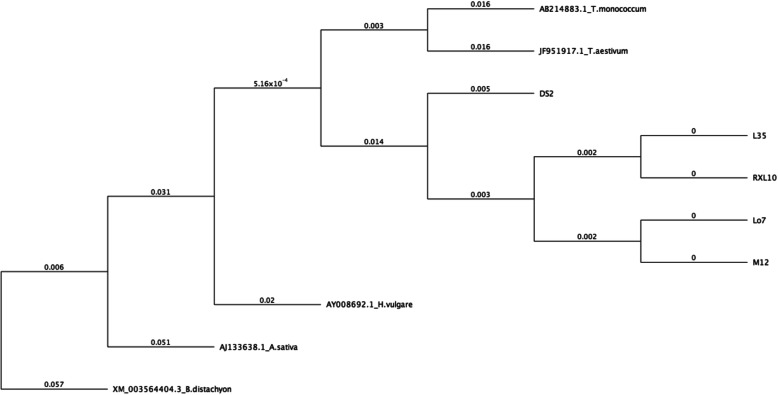
Relationships between rye inbred lines (DS2, L35, M12, RXL10, Lo7) and related species established based on *GAMYB* sequences using UPGMA method. The Lo7 sequence is sequence of scaffold Sc17168 [21]

The similarity relationships between rye inbred lines and related species (*T. aestivum*, *H. vulgare*, *A. sativa*, *B. distachyon*, *T. monococcum*) was established based on the *GAMYB* sequences (Fig. 3). As expected, the highest similarity to rye lines sequences was shown by *Triticum* species, while barley exhibited larger genetic distance, which was however smaller than that of *Avena* species. The DS2 line appeared to be the most distant line from the rest of rye inbred lines.

### Mapping of the ScGAMYB on chromosome 3R

The chromosomal location of the *ScGAMYB* has been determined in three different mapping populations: BSR, the newly derived F_2_ interline hybrid, RIL-K, and RIL-L developed previously [22]. In the BSR mapping population, 58,229 DArTseq were obtained from the genotyping by sequencing (GBS) microarray platform. For mapping purposes, 35,884 polymorphic DArTseq were assigned to the seven respective chromosomes using the information from the GBS platform (based on the markers localization reported in [21]) and the publication on the dense genetic map of rye extended with GBS markers [23]. Linkage analysis were performed on the group of DArTseqs assigned to chromosome 3R (2919) combined with DArTseqs selected from a group of loci with a previously unknown location (12,548) to which *ScGAMYB* was linked (1262).

**Fig. 4:**
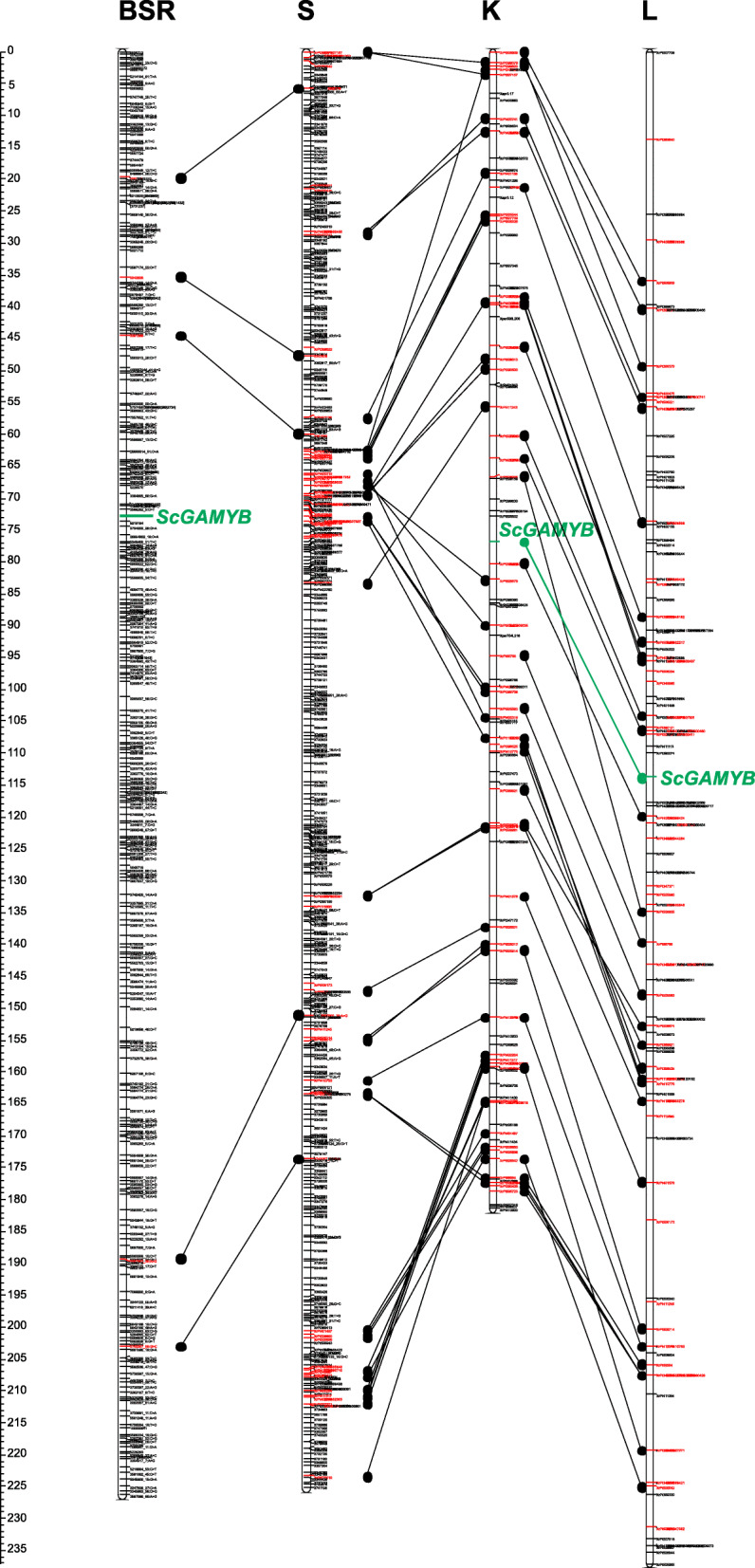
Location of the *ScGAMYB* on the chromosome 3R of populations BSR-F_2_, RIL-K [22] and RIL-L [22]. To integrate these three maps the reference map of RIL-S population [23] was used

The set of 4181 (2919 + 1262) DArTseqs and *ScGAMYB* segregation was subjected to grouping analysis. The maximum LOD value at which *ScGAMYB* remains associated with most markers was 20. After removing the loci with identical segregations, the linkage group consisted of 1843 markers and was subjected to the mapping procedure. From the set of markers linked with *ScGAMYB* in Multipoint software, 331 “delegate markers” were selected. Only the delegate markers were used as a skeleton map construction (Fig. 4, Additional file 4).

Polymorphic AS-PCR markers of *ScGAMYB* were used to access segregations within the three rye-mapping populations. The segregation ratios were consistent with monogenic model (1:1, in RIL-K and RIL-L; 3:1, in BSR-F_2_). *ScGAMYB* was mapped on the proximal part of the long arm of chromosome 3R between DArTseq-SNP 3586202_5:C > T and DArTseq-silico 5,210,184 on the BSR-F_2_ map (72.5 cM), between DArTs XrPt399424 and XrPt399390 on the RIL-K map (123.6 cM), and between XrPt390074 and XrPt507462 on the RIL-L map (108.1 cM) (Fig. 4, Additional file 4).

### Associations of ScGAMYB with AMY

This analysis was performed on two mapping populations, that were created for the PHS study and phenotyped for AMY: RIL-K [24] and RIL-L (Additional file 5). Single marker analysis (SMA) tests showed a significant relationship between the *ScGAMYB* alleles segregation and AMY variation in both populations, each in one, different vegetation season (Table 6). The third test based on CIM procedure confirmed the association of *ScGAMYB* with AMY in RIL-L population during another year of study (Table 6). The QTL underlying AMY, which peak was most closely linked to *ScGAMYB* explained 24.26% of the phenotypic variance, with LOD score of 2.71.

**Table 6:** Relationship between allele polymorphism in the *ScGAMYB* locus and variance of α-amylase activity (AMY) established using Kruskal-Wallis test (K-W), Fisher test (F) and composite interval mapping (CIM) procedure in RIL-K and RIL-L mapping populations. Only statistically significant values are shown

Population	Year	*ScGAMYB* allele	Number of genotypes	Mean AMY value		Test	Statistics	Significance
				mm	U/ml			
RIL-K	2014	a (541)	42	11.94	8.12	K-W	H = 7.24	*p* = 0.007**
		b (Ot1–3)	39	10.89	1.79			
RIL-L	2011	a (DS2)	17	15.09	72.80	F	F = 5.21	*p* = 0.025*
		b (RXL10)	25	14.33	33.60			
RIL-L	2007	a (DS2)	17	15.02	56.63	CIM	LOD = 2.71	*p* ≤ 0.050*
		b (RXL10)	23	14.44	42.73			

## Discussion

The sequence of *ScGAMYB* characterized here shows high homology to *TaGAMYB* and *HvGAMYB* in wheat and barley [12] (Table 3). The gene structure is also similar to wheat and barley orthologues having four exons and three introns where the start codon and functional MYB domain are located on exon 2 (Fig. 1). The *ScGAMYB* map position was identified on the proximal part of the long arm of chromosome 3R, a syntenic segment to *GAMYB* regions in wheat, barley [12] and rice [13]. The alignment of the *ScGAMYB* sequence to the whole genome shotgun sequence assembly of rye inbred line Lo7 [21] confirmed this location since the gDNA scaffold no. *Sc170168*, containing the entire sequence of *ScGAMYB*, was located on chromosome 3R in position of 92 cM [21].

The entire mRNA sequence of the *ScGAMYB* was identified by analyzing RNAseq data from four inbred rye lines. Due to the lack of a complete reference genome of rye, transcripts were assembled de novo using the Trinity package. We obtained over 96% bases of high quality (Table 1) for all lines. Low quality bases and adapter sequences were trimmed before further analysis. N50 statistics are widely used to assess the quality of the assembly [20]. The observed value of N50 (1366 nt) was higher than the average length of the contig (840 nt) suggesting a good assembly quality [25]. The assembly of rye sequences is associated with various challenges related to the large size of the genome, high repetition rate, and the lack of a reference genome. Although the de novo transcriptome assembly approach is beneficial, because it allows detection of new gene isoforms, as well as a better understanding of the mechanisms of alternative gene assembly in the species studied [26], there is also a risk of incomplete assembly and fragmentation of transcripts [27]. Schatz et al. [28] describes the difficulties of de novo assembling of plant transcriptomes by comparing the whole procedure with arranging the puzzle from the blue sky photo. The complications resulting from the size and complexity of plant genomes, imply the need to use large computational resources, checking the correctness of submission of readings and the use of appropriate parameters to which the assembly process is very sensitive [28]. This factors can explain the large number of transcripts in our experiment, probably overstated relative to the gene number. The analysis and assembly of the transcriptome based on 17 samples of the rye inbred lines allowed to receive almost 219,000 transcripts (Table 2). This is quite a large number of contigs, even compared to results obtained for rye in other transcriptome experiment [29]. Xu and coauthors [29] sequenced eight rye libraries using the 454 GS FLX Titanium (Roche) technology and received 120,416 rye contigs. In another transcriptome sequencing experiment using Roche technology, 115,400 contigs of five inbred winter rye lines [30] were collected. Differences in the amount of assembled transcripts can result from the use of different sequencing technologies and a different number of samples undergoing sequence reading [28].

Overestimated number of contigs in our experiment was probably the reason for the occurrence of the *ScGAMYB* sequence in two unlinked contigs. The entire mRNA sequence of the *ScGAMYB* was found in contig c81081_g3_i1 containing exon 2, 3 and 4 sequences, and contig c81081_g4_i1 having exon 1 sequence.

Of 24 polymorphisms (SNPs) found within the cds, five affected the aa composition and secondary structure of the ScGAMYB protein. The level of polymorphism detected in rye was thus higher than that reported for wheat and barley within a much wider genetic material [12]. The level of nucleotide diversity of the *ScGAMYB* sequence was determined in several cereal species based on the number of identified SNPs. There were 24 SNPs in the *ScGAMYB* cds. The *TaGAMYB* cds differs in five nucleotide positions and *HvGAMYB* sequence contains seven SNPs. This is not surprising because rye is an outcrossing species and is more heterogeneous than self-pollinated cereals. Finding SNPs that can affect the secondary structure of *ScGAMYB* can lead to development of functional markers for this key regulatory protein. Haseneyer et al. [12], analyzing barley *HvGAMYB* showed more SNPs in exon 3 than in the other exons. This observation suggested that exon 3 was suitable for finding intra-gen polymorphism and PCR-primer design. Indeed, a search for polymorphisms in exon 3 of the *ScGAMYB* allowed us to develop AS-PCR markers and identify the gene map position.

*ScGAMYB* is located within a near-centromeric region on chromosome arm 3RL, where the QTLs for AMY were found in rye [17, 24, 31]. Alpha-amylase production in cereal grain has a complex genetic basis, consisting of numerous QTLs. Studies on rye revealed as many as 16 QTLs for AMY on RIL-K and RIL-L maps [32], and 14 QTLs on RIL-M map [19]. They were detected on all seven chromosomes, of which two were identified on chromosome 3R on each map [19, 32]. Multi-genic basis of the trait means that most genes have rather low effects.

As expected, the analysis performed here indicated a statistically proved, however moderate relationship between the *ScGAMYB* allelic segregation and AMY in grain. The relationship of *ScGAMYB* and AMY revealed here comprises its function as a transcriptional activator of α-amylase structural genes in cereal grain. However, this molecular function of GAMYB may be negatively affected by interactions with a number of transcription factors such as SLN1, Vp1 and PKABA1 [8, 10, 33]. SLN1 is a negative regulator of GA responses in aleurone cells of barley (*Hordeum vulgare* L.). It is a product of the *Sln1* (*Slender1*) gene and is necessary for GAMYB repression in aleurone cells [10]. SLN1 activity in aleurone cells is post-translationally regulated. In response to GA, SLN1 levels decrease before increase of GAMYB production [10]. Studies of dominant dwarf mutants in *Sln1* indicate that GA works by regulating SLN1 degradation rather than translation [10]. The *Viviparous1* (Vp1) gene is a key component of ABA signaling identified for seed dormancy in cereals [34]. *HvVP1* transcripts accumulate in the endosperm and embryo of developing seeds in the early stages [33]. HvVP1 controls the activation of Amy6.4 in reserve mobilization after germination via GAMYB. Research on *Vp1* confirms its central role as a gene expression switch during seed maturation and germination [33].

Other elements interfere with two members of the WRKY family: ABF1, ABF2, zinc finger protein HRT, a MAK-like kinase KGM, and a DOF transcription factor BP [4, 9, 35, 36]. These factors may reduce GAMYB’s effectiveness in α-amylase induction. Despite this complex regulatory network, *ScGAMYB* is a candidate gene for at least partial control of α-amylase production in rye grain.

The differences in the effectiveness of ScGAMYB regulatory function may be due to the predicted structural change of the protein caused by a single SNP (C/G) at the 1100 position followed by aa change (S/C) at the 367 position. Both inbred lines with cysteine, 541 and DS2, were characterized as lines of high AMY and susceptible to PHS, and were therefore selected to generate mapping populations (RIL-K and RIL-L) to study these traits [17, 24, 32]. Their counterparts, Ot1–3 and RXL10 of low AMY has serine in 367 position, like the other two lines, which were not assessed concerning AMY.

Because our previous study [18] identified QTLs for plant height, thousand grain weight and awn length within map intervals containing ScGAMYB, the role of this TF can be examined also for a relationship to these traits. Further study on a pleiotropic effect for this regulatory gene can help explain its function in various aspects of plant development.

## Conclusions

This research showed the strong similarity of rye *GAMYB* sequences to its orthologues in other *Graminae* (*Triticum, Hordeum, Brachypodium, Avena*) and confirmed that the position in the genome (chromosome 3R) is consistent with the collinearity rule of cereal genomes.

Designed here sequence-based AS-PCR markers (ten pairs of primers) were used for genetic mapping purposes and can be useful in research and application purposes of rye and Triticale.

The regulatory effect of GAMYB on AMY was confirmed. This relationship depends on environmental conditions, which is typical for QTL genes. Therefore, *ScGAMYB* is a candidate gene for partial control of α-amylase production in rye grain.

The predicted structural protein change in the aa region 362–372, caused by a single SNP (C/G) at the 1100 position in *ScGAMYB* cds and single aa sequence change (S/C) at the 367 position, may cause the differences in the effectiveness of ScGAMYB regulatory function associated with AMY.

## Methods

### Plant material and mapping populations

All plant material was collected and bred for many years at the Department of Plant Genetics, Breeding and Biotechnology of the West-Pomeranian University of Technology, Szczecin. All lines of winter rye used were advanced in inbred (at least S_10_). This material has not been deposited in a publicly available herbarium.

Four rye inbred lines of different pedigrees were chosen to the transcriptome sequencing analyzes: DS2, RXL10, L35 and M12. DS2 is derived from *S. dighoricum* × *S. cereale* cv Smolickie; the ancestor of line RXL10 is *S. cereale* cv Zeelandzkie; L35 and M12 are recombinant inbred lines (RIL) derived from the two different interline hybrids: DS2 × RXL10 and S120N/95 × S76N/95, respectively.

For genetic mapping, different pairs of parental inbred lines were screened to identify polymorphism at the *ScGAMYB* locus using AS-SCAR. Three mapping population were selected: BSR, RIL-K and RIL-L. BSR is the new F_2_ mapping population obtained from a cross between inbred lines S32N/07 and RXL10. The S32N/07 inbred line is semi-tall, with narrow and rolling leaves, early flowering and was developed and provided by Danko Plant Breeding Ltd. (Choryń, Poland) for research purposes. The RXL10 inbred line is dwarf, with wide and straight leaves, late flowering. RIL-K is a population of recombinant inbred lines derived from the 541 (high, sensitive to PHS, high AMY, early flowering) × Ot1–3 (short, resistant to PHS, low AMY, late flowering) intercross [22]. The origin of the 541 line is described as KaH6 × [(MS69–8-1 × *S. cereale* cv Smolickie) F_2_MS × KaH]F_1_MP; the Ot1–3 line originates from Swedish cv Otello. RIL-L is a population of recombinant inbred lines derived from the DS2 (high, sensitive to PHS, high AMY, early flowering) × RXL10 intercross.

### Phenotyping analyzes of AMY

The trials were conducted on experimental fields of the West Pomeranian University of Technology in Szczecin (53.45°N, 14.53°E). Each line of the 82 RILs-K population was represented by 33 plants grown in a randomized block design over 3 replicates in 2013 and 2014. 74 lines of RIL-L population was represented by 24 plants grown in a randomized block design over 2 replicates in 2005, 2007 and 2011 (Additional file 5). Here, 1.5 g of mature kernels from each RIL and each replicate were milled and AMY in the grain was assessed using the gel diffusion method [37].

### DNA isolation

Genomic DNA was extracted using DNeasy Plant Mini Kit (Qiagen) from leaves of two-week-old seedlings. The samples were frozen at − 80 °C or lyophilized at − 56 °C in Alpha 1–2 LD plus Lyophilizer and stirred into powder in a Retsch MM200 mill. The concentration of DNA was established in an EPOCH (Biotek) spectrophotometer. DNA samples were equilibrated to 10 ng/μl.

### ScGAMYB sequence analysis

The *ScGAMYB* rye sequences were identified in RNA-seq libraries created for four rye inbred lines: DS2, RXL10, L35, and M12. Each line was represented by 4–6 biological repetitions. Total RNA was purified from six individuals of line DS2 and M12 and four individuals of line RXL10 and L35. TRI Reagent Kit (Sigma-Aldrich) was used for RNA purification. After isolation, 20 samples of total RNA were sent to Macrogen (South Korea) for performing further stages of the RNAseq procedure. RNA quality was assessed using RIN parameter (RNA integrity number). Samples for which the RIN value was below 5.5 were excluded from further analysis.

The mRNA in total RNA was converted into a library of template molecules suitable for subsequent cluster generation using the reagents provided in the Illumina® TruSeq™ RNA Sample Preparation Kit. First, the poly-A containing mRNA molecules using poly-T oligo-attached magnetic beads were purified. The mRNA was fragmented into small pieces using divalent cations under elevated temperature. The cleaved RNA fragments were copied into the first strand of cDNA using reverse transcriptase and random primers. These cDNA fragments went through an end repair process, the addition of a single ‘A’ base, and then ligation of the adapters. The products were then purified and enriched by PCR to create the final cDNA library. Sequencing was performed on an Illumina HiSeq2000 based on the Solexa’s Sequencing-by-Synthesis method. Illumina reads of all samples had been submitted to the Sequence Read Archive at the National Center for Biotechnology Information (http://www.ncbi.nlm.nih.gov/sra), under accession numbers from SRX2636904 to SRX2636920, under BioProject PRJNA371298 (ID: 371298).

Sequence quality control was performed at FastQC software (http://www.bioinformatics.babraham.ac.uk/projects/fastqc). Trimmomatic (0.32) (http://www.usadellab.org/cms/?page=trimmomatic) - multithreaded command line tool was used to trim and crop Illumina (FASTQ) data as well as to remove adapters. De novo reconstruction of transcriptomes from RNA-seq data was performed using Trinity (http://trinityrnaseq.sourceforge.net) [26]. RSEM (1.2.15) software (http://deweylab.biostat.wisc.edu/rsem/) tool was used for quantifying transcript abundances from RNAseq data. Transcripts annotation based on BlastX bioinformatic tool (http://blast.ncbi.nlm.nih.gov/Blast.cgi? CMD = Web&PAGE_TYPE = BlastDocs&DOC_TYPE = ProgSelectionGuide) was done. This program compares the six-frame conceptual translation products of a nucleotide query sequence (both strands) against a protein sequence database (Gene Ontology, EGGNOG). Finally, the contigs covering the entire cds of *ScGAMYB* was revealed using Geneious software version 10.2.4. The same software was used to map the fragment of *ScGAMYB* to the whole genome shotgun sequence assembly of rye inbred line Lo7 [21]. EMBOSS package 6.5.7 plug in Geneious software was applied for protein structure prediction. This approach allowed verification of the gene structure and its chromosomal location.

### SNPs identification

Identification of SNPs within the cds of the *ScGAMYB* of six rye lines was carried out with the Geneious software version 10.2.4. From the readings deposited in the SRA database, those that covered the contigs containing the sequences of the described gene were selected. The reading sequences from the RXL, DS2 and L35 lines were then aligned to the sequences of the assembled contigs of the M12 line to identify point mutation sites. The sequences of lines 541 and Ot1–3 were also aligned to the sequence of contigs assembled for the M12 line. The SNPs were described by their position in the nucleotide sequence and the type of mutation. It was then checked whether the mutation changed the type of aa in the protein sequence.

### Gene amplification, cloning and sequencing

Sequences of the primers used for the amplification of *ScGAMYB* in the inbred lines 541 and Ot1–3 (F: 5′-tatgcaccacagctttcagc-3′, R: 5′-gtgcaggaggaattttggag-3′) were derived from the conserved sequences of the third exon of *ScGAMYB* using Primer 3 and Oligo 7 computer programs. Amplifications were performed in a 20 μl reaction volume containing: 1 × Phusion HF Buffer, 200 μM of each dNTP, 5 pmol of each primer, 0.4 U of Phusion HotStart II Polymerase (Finnzymes) and 30 ng of DNA template. Amplification was carried out in a BioRad Thermal Cycler. The following thermal profile was used: initial denaturation at 98 °C for 30 s, 10 cycles of denaturation at 98 °C for 10 s, primers annealing at 60 °C − 1 °C per cycle for 15 s, extension at 72 °C for 20 s, and 25 cycles of denaturation at 98 °C for 10 s. The primers annealed at 50 °C for 15 s with extension at 72 °C for 20 s; the final extension was at 72 °C for 300 s.

PCR products were separated in a 1.5% agarose gel (Serva-Molecular Biology Grade). The approximately 770 nt long monomorphic rye amplicons from lines 541 and Ot1–3 were cut off the gel using a sterile extractor from Promega and eluted using MiniElute Gel Extraction Kit (Qiagen). Amplification products were cloned using ligation into pCR2.1-TOPO vectors in the presence of topoisomerase I and transformation of chemically competent *E.coli* cells via the heat shock method (Invitrogen).

Plasmids were isolated using a Plasmid Mini Kit (A&A Biotechnology, BLIRT). Genome Lab DTCS reagents (Beckman Coulter) were used to sequencing of PCR products. PCR products were cleaned by magnetic beads (Agencourt CleanSEQ, Beckman Coulter) and sequenced in a Beckman Coulter CEQ 8000 Genetic Analysis System. Sequencing was carried out for both strands by Sanger method, which offered a consensus sequence of gene fragments via Geneious 10.2.4. computer program [38].

### AS-PCR for detecting polymorphisms in ScGAMYB sequence

Identification of SNPs between the *ScGAMYB* clones from Ot1–3 and 541 parental lines allowed the design of primers for allele-specific PCR (AS-PCR) using Oligo7 software. Three types of primers were designed to detect individual SNPs. The first type (forward, F or reverse, R) was specifically complementary to the allele sequence from line Ot1–3. The second kind of primer (F or R) was specific to the sequence of the line 541 allele, and the third one (F or R) was complementary to the sequences common for both lines (Table 5).

Differentiating nucleotides in this assay were located in the last pentamer at the 3′ terminal base of the primers. The allele specific products were amplified in PCR mix in 12.5 μl containing: 1 × SNPase buffer, 93.75 μM of each dNTP, 0.775 mM MgCl_2_, 2.5 pmol of each primer, 0.5 U of SNPase HotStart polymerase (Bioron), and 15 ng of DNA template.

Amplification was performed using a BioRad Thermal Cycler. The cycling parameters were 94 °C for 60 s of pre-denaturation followed by 35 cycles of 94 °C for 30 s, 60 °C for 15 s, 72 °C for 30 s, and a final extension at 72 °C for 300 s.

### Genetic mapping

*The ScGAMYB* was genetically mapped on a new map of S32N/07 × RXL10-F_2_ population (BSR), consisting of the DArTseqs and on the 541 × Ot1–3 (RIL-K) and DS2 × RXL10 (RIL-L) high-density DArT-based maps developed previously [22].

DNA of the 92 plants of BSR-F_2_ generation and two parental lines were sent to the external institution (Diversity Arrays Technology Pty Ltd., Canberra, Australia) for GBS analysis (http://www.diversityarrays.com/) [39].

DArT [40] is cost-effective sequence-independent ultra-high-throughput marker system. DArT develops markers through a microarray hybridization method and can produce thousands of polymorphic loci in a single assay. DArTseq is a GBS system which utilizes next generation sequencing (NGS) platforms (Illumina, USA) to sequence the most informative representations of genomic DNA samples to help marker discovery [41]. In comparison to the array version of DArT, DArTseq results in higher marker densities and two types of data are generated: SilicoDArT and SNP [41]. SilicoDArTs are microarray markers that are dominant and scored for the presence or absence of a single allele (restriction fragment). DArTseq based SNPs are co-dominant markers utilizing differences in the sequence of restriction fragments.

The segregations of DArTseqs in BSR mapping population were applied for mapping the *ScGAMYB* locus. The JoinMap5 package [42] was used to establish a group of markers linked to the *ScGamyb* (grouping tree command) and to remove identical loci to simplify the mapping procedure. The genetic map construction was conducted using Multipoint 3.2 software [43]. The group of DARTseq markers segregating in accordance to “b, d” (*GAMYB* segregation) and “a, h, b” schemes were used to construct the genetic map. The “order” command was applied for marker linkage groups formed at a maximum threshold level of recombination frequencies at 0.005. The “control of monotony” command was used for detection and removing problematic markers that caused neighborhood instabilities. Finally, the ordering was repeated with the Kosambi mapping function and a genetic map was generated.

The genetic maps of the RIL-K and RIL-L populations were constructed previously using classic DArTs and PCR markers [22]. Segregation of the *ScGAMYB* was added to the set of RIL-K and RIL-L markers and remapped. The mapping procedure applied Multipoint 3.2 software as with the BSR population map. To reduce the inflation of genetic distances on a high-density genetic map, the average length of the consensus map [22] was used for scaling obtained linkage groups as previously described [44, 45]. A genetic map of population RIL-S [23] was used as a reference map to rescale the distances between loci. The RIL-S map consisted of both DArT and DArTseq markers, which allowed to compare the position of the *ScGAMYB* on three analyzed maps using MapChart software [46].

### Statistical analysis and QTL mapping

The statistical relationship between the segregation of molecular markers and AMY was analyzed with the nonparametric Kruskal–Wallis’ test using STATISTICA v. 12.0 software (http://www.statsoft.com), and by QTL mapping performed using SMA based on Fisher’s test and CIM procedure with the software package WinQTL Cartographer 2.5 [47], assuming significance levels *p* ≤ 0.05.

## Supplementary information

**Additional file 1 **The characteristic and annotation data of the contig c81081 (*ScGAMYB* transcript).

**Additional file 2 **Coding sequence of *ScGAMYB* and SNPs differentiating six rye inbred lines.

**Additional file 3 **Amino acid (aa) composition and polymorphisms of the protein translated from the *ScGAMYB*. Yellow backlight means differences in aa, pink backlight indicates changes that are important for the 2D protein structure and related to the effect of ScGAMYB on AMY.

**Additional file 4 **1: The genetic map of chromosome 3R with *ScGAMYB* of three rye mapping populations: BSR-F_2_, RIL-K, and RIL-L. 2: The segregations of markers from chromosome 3R in rye mapping population BSR-F_2._ 3: The segregations of markers from chromosome 3R in rye mapping population RIL-K. 4: The segregations of markers from chromosome 3R in rye mapping population RIL-L.

**Additional file 5.** Alpha-amylase activity in rye mapping population RIL-L.

## Data Availability

The mapping data are submitted as the supplementary material Additional file 4_4 and are available in the article by Milczarski [21, 38]. The raw transcripts sequences data for rye inbred lines are deposited in Sequence Read Archive (SRA), in GeneBank (NCBI) under BioProject No PRJNA371298 (ID: 371298, accessions from SRX2636904 to SRX2636920).

## Abbreviations

aa: amino acid
ABA: abscisic acid
AMY: α-amylase activity
AS: allele-specific
cds: coding sequence
CIM: composite interval mapping
DArT: diversity array technology
GA: gibberellin
nt: nucleotide
GBS: genotyping by sequencing
LOD: logarithm of the odds ratio
PHS: pre-harvest sprouting
QTL: quantitative trait locus
RIL: recombinant inbred line
SCAR: sequence characterized amplified region
SNP: single nucleotide polymorphism
SMA: single marker analysis
TF: transcription factor
