# Supplementary material for: The GAMYB gene in rye: sequence, polymorphisms, map location, allele-specific markers, and relationship with α-amylase activity
Source: BMC Genomics. 2020 Aug 24;21:578. doi: 10.1186/s12864-020-06991-3 (PMC7444254; doi:10.1186/s12864-020-06991-3)
Supplement: Supplementary file 2 — Additional file 2 Coding sequence of ScGAMYB and SNPs differentiating six rye inbred lines. [file 12864_2020_6991_MOESM2_ESM.docx]

Additional file 2. Coding sequence of *ScGAMYB* and SNPs differentiating six rye inbred lines.

1 10 20 30 40 50 60

| | | | | | |

*ScGAMYB* M12 ATGTACCGGGTGAAGAGCGAGAGCGACTGCGAGATGATGCATCAGGAGGACCAGATGGAC

*ScGAMYB* DS2 ATGTACCGGGTGAAGAGCGAGAGCGACTGCGAGATGATGCATCAGGAGGACCAGATGGAC

*ScGAMYB* RXL10 ATGTACCGGGTGAAGAGCGAGAGCGACTGCGAGATGATGCATCAGGAGGACCAGATGGAC

*ScGAMYB* L35 ATGTACCGGGTGAAGAGCGAGAGCGACTGCGAGATGATGCATCAGGAGGACCAGATGGAC

*ScGAMYB* 541 ------------------------------------------------------------

*ScGAMYB* Ot1-3 ------------------------------------------------------------

*ScGAMYB* M12 TCGCCGGTGGGCGACGACGGCAGCAGCGGAGGGTCGCCCCACAGGGGCGGCGGGCCGCCT

*ScGAMYB* DS2 TCGCCGGTGGGCGACGACGGCAGCAGCGGAGGGTCGCCTCACAGGGGCGGCGGGCCGCCT

*ScGAMYB* RXL10 TCGCCGGTGGGCGACGACGGCAGCAGCGGAGGGTCGCCCCACAGGGGCGGCGGGCCGCCT

*ScGAMYB* L35 TCGCCGGTGGGCGACGACGGCAGCAGCGGAGGGTCGCCCCACAGGGGCGGCGGGCCGCCT

*ScGAMYB* 541 ------------------------------------------------------------

*ScGAMYB* Ot1-3 ------------------------------------------------------------

*ScGAMYB* M12 CTGAAGAAGGGGCCCTGGACGTCGGCGGAGGACGCCATCCTGGTGGACTACGTGAAGAAG

*ScGAMYB* DS2 CTGAAGAAAGGGCCCTGGACGTCGGCGGAGGACGCCATCCTGGTGGACTACGTGAAGAAG

*ScGAMYB* RXL10 CTGAAGAAGGGGCCCTGGACGTCGGCGGAGGACGCCATCCTGGTGGACTACGTGAAGAAG

*ScGAMYB* L35 CTGAAGAAGGGGCCCTGGACGTCGGCGGAGGACGCCATCCTGGTGGACTACGTGAAGAAG

*ScGAMYB* 541 ------------------------------------------------------------

*ScGAMYB* Ot1-3 ------------------------------------------------------------

*ScGAMYB* M12 CACGGCGAGGGGAACTGGAACGCCGTGCAGAAGAACACCGGGCTGTTCCGGTGCGGCAAG

*ScGAMYB* DS2 CACGGCGAGGGGAACTGGAACGCGGTGCAGAAGAACACCGGGCTGAACCGGTGCGGCAAG

*ScGAMYB* RXL10 CACGGCGAGGGGAACTGGAACGCCGTGCAGAAGAACACCGGGCTGAACCGGTGCGGCAAG

*ScGAMYB* L35 CACGGCGAGGGGAACTGGAACGCCGTGCAGAAGAACACCGGGCTGAACCGGTGCGGCAAG

*ScGAMYB* 541 ------------------------------------------------------------

*ScGAMYB* Ot1-3 ------------------------------------------------------------

*ScGAMYB* M12 AGCTGCCGCCTCCGGTGGGCGAACCACCTCAGGCCCAACCTCAAGAAGGGGGCCTTCACC

*ScGAMYB* DS2 AGCTGCCGCCTCCGGTGGATCAACTACCTCCGGCCCGACCTCAAGAAGGGGGCCTTCACC

*ScGAMYB* RXL10 AGCTGCCGCCTCCGGTGGATCAACTACCTCCGGCCCGACCTCAAGAAGGGGGCCTTCACC

*ScGAMYB* L35 AGCTGCCGCCTCCGGTGGATCAACTACCTCCGGCCCGACCTCAAGAAGGGGGCCTTCACC

*ScGAMYB* 541 ------------------------------------------------------------

*ScGAMYB* Ot1-3 ------------------------------------------------------------

*ScGAMYB* M12 CCCGAGGAGGAGAGGCTCATCATCCAGCTCCACTCCAAGATGGGCAACAAGTGGGCTCGG

*ScGAMYB* DS2 CCCGAGGAGGAGAGGCTCATCATCCAGCTCCACTCCAAGATGGGCAACAAGTGGGCTCGG

*ScGAMYB* RXL10 CCCGAGGAGGAGAGGCTCATCATCCAGCTCCACTCCAAGATGGGCAACAAGTGGGCTCGG

*ScGAMYB* L35 CCCGAGGAGGAGAGGCTCATCATCCAGCTCCACTCCAAGATGGGCAACAAGTGGGCTCGG

*ScGAMYB* 541 ------------------------------------------------------------

*ScGAMYB* Ot1-3 ------------------------------------------------------------

*ScGAMYB* M12 ATGGCCGCTCATTTGCCAGGGCGTACTGACAATGAAATAAAGAATTACTGGAACACTCGA

*ScGAMYB* DS2 ATGGCCGCTCATTTGCCAGGGCGTACTGACAATGAAATAAAGAATTACTGGAACACTCGA

*ScGAMYB* RXL10 ATGGCCGCTCATTTGCCAGGGCGTACTGACAATGAAATAAAGAATTACTGGAACACTCGA

*ScGAMYB* L35 ATGGCCGCTCATTTGCCAGGGCGTACTGACAATGAAATAAAGAATTACTGGAACACTCGA

*ScGAMYB* 541 ------------------------------------------------------------

*ScGAMYB* Ot1-3 ------------------------------------------------------------

*ScGAMYB* M12 ATAAAGAGATGTCAGCGAGCCGGCTTGCCAGTATATCCTGCTAGTGTATGCAATCAATCT

*ScGAMYB* DS2 ATAAAGAGATGTCAGCGAGCCGGCTTGCCAGTATATCCTGCTAGTGTATGCAATCAATCT

*ScGAMYB* RXL10 ATAAAGAGATGTCAGCGAGCCGGCTTGCCAGTATATCCTGCTAGTGTATGCAATCAATCT

*ScGAMYB* L35 ATAAAGAGATGTCAGCGAGCCGGCTTGCCAGTATATCCTGCTAGTGTATGCAATCAATCT

*ScGAMYB* 541 ------------------------------------------------------------

*ScGAMYB* Ot1-3 ------------------------------------------------------------

*ScGAMYB* M12 TCAAATGAAGATCAGCAGGGCTCCAGCGATTTCAACTGCGGCGAAAATCTTTCCAGTGAC

*ScGAMYB* DS2 TCAAATGAAGATCAGCAGGGCTCCAGCGATTTCAACTGCGGCGAAAATCTTTCCAGTGAC

*ScGAMYB* RXL10 TCAAATGAAGATCAGCAGGGCTCCAGCGATTTCAACTGCGGCGAAAATCTTTCCAGTGAC

*ScGAMYB* L35 TCAAATGAAGATCAGCAGGGCTCCAGCGATTTCAACTGCGGCGAAAATCTTTCCAGTGAC

*ScGAMYB* 541 ------------------------------------------------------------

*ScGAMYB* Ot1-3 ------------------------------------------------------------

*ScGAMYB* M12 CTTCTGAATGGAAATGGTCTTTACCTGCCAGATTTTACCTGCGACAATTTCATTGCTAAT

*ScGAMYB* DS2 CTTCTGAATGGAAATGGTCTTTATCTGCCAGATTTTACCTGCGACAATTTCATTGCTAAT

*ScGAMYB* RXL10 CTTCTGAATGGAAATGGTCTTTACCTGCCAGATTTTACCTGCGACAATTTCATTGCTAAT

*ScGAMYB* L35 CTTCTGAATGGAAATGGTCTTTACCTGCCAGATTTTACCTGCGACAATTTCATTGCTAAT

*ScGAMYB* 541 ------------------------------------------------------------

*ScGAMYB* Ot1-3 ------------------------------------------------------------

*ScGAMYB* M12 TCAGAGGCTTTATCTTATGCACCACAGCTTTCAGCTGTTTCAATAAGCAGTTTGCTTGGC

*ScGAMYB* DS2 TCAGAGGCTTTATCTTATGCACCACAGCTTTCAGCTGTTTCAATAAGCAGTTTGCTTGGC

*ScGAMYB* RXL10 TCAGAGGCTTTATCTTATGCACCACAGCTTTCAGCTGTTTCAATAAGCAGTTTGCTTGGC

*ScGAMYB* L35 TCAGAGGCTTTATCTTATGCACCACAGCTTTCAGCTGTTTCAATAAGCAGTTTGCTTGGC

*ScGAMYB* 541 ---------------TATGCACCACAGCTTTCAGCTGTTTCAATAAGCAGTTTGCTTGGC

*ScGAMYB* Ot1-3 ---------------TATGCACCACAGCTTTCAGCTGTTTCAATAAGCAGTTTGCTTGGC

*ScGAMYB* M12 CAGAGCTTTGCATCCAAAAATTGCGGCTTCATGGATCCAGTAAACCAAGCAGGGATGCTG

*ScGAMYB* DS2 CAGAGCTTTGCATCCAAAAACTGCGGCTTCATGGATCAAGTAAACCAAGCAGGGATGCTA

*ScGAMYB* RXL10 CAGAGCTTTGCATCCAAAAATTGCGGCTTCATGGATCCAGTAAACCAAGCAGGGATGCTG

*ScGAMYB* L35 CAGAGCTTTGCATCCAAAAATTGCGGCTTCATGGATCCAGTAAACCAAGCAGGGATGCTG

*ScGAMYB* 541 CAGAGCTTTGCATCCAAAAACTGCGGCTTCATGGATCAAGTAAACCAAGCAGGGATGCTA

*ScGAMYB* Ot1-3 CAGAGCTTTGCATCCAAAAACTGCGGCTTCATGGATCAAGTAAACCAAGCAGGGATGCTA

*ScGAMYB* M12 AAACAGTCTGACCCATTACTTCCTGGATTGAGCGACACCATCAATGGGGCGCTCTCCTCG

*ScGAMYB* DS2 AAACAGTCTGACCCGTTACTCCCTGGATTGAGCGACACCATCAATGGGGCGCTCTCCTCG

*ScGAMYB* RXL10 AAACAGTCTGACCCATTACTTCCTGGATTGAGCGACACCATCAATGGGGCGCTCTCCTCG

*ScGAMYB* L35 AAACAGTCTGACCCATTACTTCCTGGATTGAGCGACACCATCAATGGGGCGCTCTCCTCG

*ScGAMYB* 541 AAACAGTCTGACCCGTTACTCCCTGGATTGAGCGACACCATCAATGGGGCGCTCTCCTCG

*ScGAMYB* Ot1-3 AAACAGTCTGACCCGTTACTCCCTGGATTGAGCGACACCATCAATGGGGCGCTCTCCTCG

*ScGAMYB* M12 GTCGATCAGTTCTCAAATGACTCTGAGAAGCTCAAGCAGGCTCTGGGTTTTGACTATCTC

*ScGAMYB* DS2 GTCGATCAGTTCTCAAATGACTCTGAGAAGCTCAAGCAGGCTCTGGGTTTTGACTATCTC

*ScGAMYB* RXL10 GTCGATCAGTTCTCAAATGACTCTGAGAAGCTCAAGCAGGCTCTGGGTTTTGACTATCTC

*ScGAMYB* L35 GTCGATCAGTTCTCAAATGACTCTGAGAAGCTCAAGCAGGCTCTGGGTTTTGACTATCTC

*ScGAMYB* 541 GTCGATCAGTTCTCAAATGACTCTGAGAAGCTCAAGCAGGCTCTGGGTTTTGACTATCTC

*ScGAMYB* Ot1-3 GTCGATCAGTTCTCAAATGACTCTGAGAAGCTCAAGCAGGCTCTGGGTTTTGACTATCTC

*ScGAMYB* M12 CACGAAGCCAACTCTAGCAGCAAGATTATTGCACCATTTGGGGGTGCACTTACTGGCAGC

*ScGAMYB* DS2 CACGAAGCCAACTCTAGCAGCAAGATTATTGCACCATTTGGGGGTGCACTTACTGGCAGC

*ScGAMYB* RXL10 CACGAAGCCAACTCTAGCAGCAAGATTATTGCACCATTTGGGGGTGCACTTACTGGCAGC

*ScGAMYB* L35 CACGAAGCCAACTCTAGCAGCAAGATTATTGCACCATTTGGGGGTGCACTTACTGGCAGC

*ScGAMYB* 541 CACGAAGCCAACTCTAGCAGCAAGATTATTGCACCATTTGGGGGTGCACTTACTGGCAGC

*ScGAMYB* Ot1-3 CACGAAGCCAACTCTAGCAGCAAGATTATTGCACCATTTGGGGGTGCACTTACTGGCAGC

*ScGAMYB* M12 CATGCCTTTTTAAATGGCACCTTCTCTACTTCTAGGACCATCAATGGTCCTTTGAAGATG

*ScGAMYB* DS2 CATGCCTTTTTAAATGGCACCTTCTCTACTTCTAGGACCATCAGTGGTCCTTTGAAGATG

*ScGAMYB* RXL10 CATGCCTTTTTAAATGGCACCTTCTCTACTTCTAGGACCATCAATGGTCCTTTGAAGATG

*ScGAMYB* L35 CATGCCTTTTTAAATGGCACCTTCTCTACTTCTAGGACCATCAATGGTCCTTTGAAGATG

*ScGAMYB* 541 CATGCCTTTTTAAATGGCACCTTCTCTACTTCTAGGACCATCAGTGGTCCTTTGAAGATG

*ScGAMYB* Ot1-3 CATGCCTTTTTAAATGGCACCTTCTCTACTTCTAGGACCATCAGTGGTCCTTTGAAGATG

*ScGAMYB* M12 GAGCTCCCTTCACTCCAAGATACCGAATCTGATCCGAATAGCTGGCTCAAGTATACCGTG

*ScGAMYB* DS2 GAGCTCCCTTCACTCCAAGATACCGAATCTGATCCGAATAGCTGGCTCAAGTATACCGTG

*ScGAMYB* RXL10 GAGCTCCCTTCACTCCAAGATACCGAATCTGATCCGAATAGCTGGCTCAAGTATACCGTG

*ScGAMYB* L35 GAGCTCCCTTCACTCCAAGATACCGAATCTGATCCGAATAGCTGGCTCAAGTATACCGTG

*ScGAMYB* 541 GAGCTCCCTTCACTCCAAGATACCGAATCTGATCCGAATAGCTGGCTCAAGTATACCGTG

*ScGAMYB* Ot1-3 GAGCTCCCTTCACTCCAAGATACCGAATCTGATCCGAATAGCTGGCTCAAGTATACCGTG

*ScGAMYB* M12 GCTCCTGCGATGCAGCCTACGGAGTTGGTTGATCCCTACCTGCAGTCCCCGACAGCAACC

*ScGAMYB* DS2 GCTCCTGCGATGCAGCCTACGGAGTTGGTTGATCCCTACCTGCAGTCCCCGACAGCAACC

*ScGAMYB* RXL10 GCTCCTGCGATGCAGCCTACGGAGTTGGTTGATCCCTACCTGCAGTCCCCGACAGCAACC

*ScGAMYB* L35 GCTCCTGCGATGCAGCCTACGGAGTTGGTTGATCCCTACCTGCAGTCCCCGACAGCAACC

*ScGAMYB* 541 GCTCCTGCGATGCAGCCTACGGAGTTGGTTGATCCCTACCTGCAGTCCCCGACAGCAACC

*ScGAMYB* Ot1-3 GCTCCTGCGATGCAGCCTACGGAGTTGGTTGATCCCTACCTGCAGTCCCCGACAGCAACC

*ScGAMYB* M12 CCGTCAGTGAAATCGGAGTCTGCGTCGCCGAGGAACAGCGGCCTCTTGGAAGAGCTGCTT

*ScGAMYB* DS2 CCGTCAGTGAAATCGGAGTGTGCGTCGCCCAGGAACAGCGGCCTCTTGGAAGAGCTGCTT

*ScGAMYB* RXL10 CCGTCAGTGAAATCGGAGTCTGCGTCGCCGAGGAACAGCGGCCTCTTGGAAGAGCTGCTT

*ScGAMYB* L35 CCGTCAGTGAAATCGGAGTCTGCGTCGCCGAGGAACAGCGGCCTCTTGGAAGAGCTGCTT

*ScGAMYB* 541 CCGTCAGTGAAATCGGAGTGTGCGTCGCCCAGGAACAGCGGCCTCTTGGAAGAGCTGCTT

*ScGAMYB* Ot1-3 CCGTCAGTGAAATCGGAGTCTGCGTCGCCGAGGAACAGCGGCCTCTTGGAAGAGCTGCTT

*ScGAMYB* M12 CATGAAGCTCAGGGACTAAGATCTGGGAAGAATCAGCAGCTTTCCGTGAGGAGTTCAAGT

*ScGAMYB* DS2 CATGAAGCTCAGGGACTAAGATCTGGGAAGAATCAGCAGCTTTCTGTGAAGAGTTCAAGT

*ScGAMYB* RXL10 CATGAAGCTCAGGGACTAAGATCTGGGAAGAATCAGCAGCTTTCCGTGAGGAGTTCAAGT

*ScGAMYB* L35 CATGAAGCTCAGGGACTAAGATCTGGGAAGAATCAGCAGCTTTCCGTGAGGAGTTCAAGT

*ScGAMYB* 541 CATGAAGCTCAGGGACTAAGATCTGGGAAGAATCAGCAGCTTTCTGTGAGAAGTTCAAGT

*ScGAMYB* Ot1-3 CATGAAGCTCAGGGACTAAGATCTGGGAAGAATCAGCAGCTTTCCGTGAGGAGTTCAAGT

*ScGAMYB* M12 TCCTCTGTCAGCACGCCGTGTGATACTACGGTGGTTAGCCCAGAGTTTGATCTCTGTCAG

*ScGAMYB* DS2 TCCTCTGTCAGCACGCCGTGTGATACTACGGTGGTTAGCCCAGAGTTTGATCTCTGTCAG

*ScGAMYB* RXL10 TCCTCTGTCAGCACGCCGTGTGATACTACGGTGGTTAGCCCAGAGTTTGATCTCTGTCAG

*ScGAMYB* L35 TCCTCTGTCAGCACGCCGTGTGATACTACGGTGGTTAGCCCAGAGTTTGATCTCTGTCAG

*ScGAMYB* 541 TCCTCTGTCAGCACGCCGTGTGATACTACGGTGGTTAGCCCAGAGTTTGATCTCTGTCAG

ScGAMYB Ot1-3 TCCTCTGTCAGCACGCCGTGTGATACTACGGTGGTTAGCCCAGAGTTTGATCTCTGTCAG

*ScGAMYB* M12 GAATATTGGGAAGAACGTCTGAATGAGTATGCTCCATTCAGTGGTAATTCACTCACTGGA

*ScGAMYB* DS2 GAATATTGGGAAGAACGTCTGAATGAGTATGCTCCATTCAGTGGTAATTCACTCACTGGA

*ScGAMYB* RXL10 GAATATTGGGAAGAACGTCTGAATGAGTATGCTCCATTCAGTGGTAATTCACTCACTGGA

*ScGAMYB* L35 GAATATTGGGAAGAACGTCTGAATGAGTATGCTCCATTCAGTGGTAATTCACTCACTGGA

*ScGAMYB* 541 GAATATTGGGAAGAACGTCTGAATGAGTATGCTCCATTCAGTGGTAATTCACTCACTGGA

*ScGAMYB* Ot1-3 GAATATTGGGAAGAACGTCTGAATGAGTATGCTCCATTCAGTGGTAATTCACTCACTGGA

*ScGAMYB* M12 TCCACCGCTCCTGTGAGCACTGCGTCGCCTGATGTTTTTCAGCTCTCCAAAATTTCTCCT

*ScGAMYB* DS2 TCCACCGCTCCTGTGAGCACTGCGTCGCCTGATGTTTTTCAGCTCTCCAAAATTTCTCCT

*ScGAMYB* RXL10 TCCACCGCTCCTGTGAGCACTGCGTCGCCTGATGTTTTTCAGCTCTCCAAAATTTCTCCT

*ScGAMYB* L35 TCCACCGCTCCTGTGAGCACTGCGTCGCCTGATGTTTTTCAGCTCTCCAAAATTTCTCCT

*ScGAMYB* 541 TCCACCGCTCCTGTGAGCACTGCGTCGCCTGATGTTTTTCAGCTCTCCAAAATTCCTCCT

*ScGAMYB* Ot1-3 TCCACCGCTCCTGTGAGCACTGCGTCGCCTGATGTTTTTCAGCTCTCCAAAATTCCTCCT

*ScGAMYB* M12 GCACAAAGCCCTTCGCTGGGATCTGGAGAGCAGGCAATGGAGCCTGCATACGAGCTTGGG

*ScGAMYB* DS2 GCACAAAGCCCTTCGCTGGGATCTGGAGAGCAGGCAATGGAGCCTGCATACGAGCTTGGG

*ScGAMYB* RXL10 GCACAAAGCCCTTCGCTGGGATCTGGAGAGCAGGCAATGGAGCCTGCATACGAGCTTGGG

*ScGAMYB* L35 GCACAAAGCCCTTCGCTGGGATCTGGAGAGCAGGCAATGGAGCCTGCATACGAGCTTGGG

*ScGAMYB* 541 GCAC--------------------------------------------------------

*ScGAMYB* Ot1-3 GCAC--------------------------------------------------------

*ScGAMYB* M12 GCTGGGGACACTTCATCTCATCCTGAAAACTTGAGGCCAGACGCATTCTTCTCCGGGAAC

*ScGAMYB* DS2 GCTGGGGACACTTCATCTCATCCTGAAAACTTGAGGCCAGACGCATTCTTCTCCGGGAAC

*ScGAMYB* RXL10 GCTGGGGACACTTCATCTCATCCTGAAAACTTGAGGCCAGACGCATTCTTCTCCGGGAAC

*ScGAMYB* L35 GCTGGGGACACTTCATCTCATCCTGAAAACTTGAGGCCAGACGCATTCTTCTCCGGGAAC

*ScGAMYB* 541 ------------------------------------------------------------

*ScGAMYB* Ot1-3 ------------------------------------------------------------

*ScGAMYB* M12 ACAACCGACTCGTCCGTCTTCAACAACGCCATAGCTATGCTCCTGGGCAACGACATGAAC

*ScGAMYB* DS2 ACAACCGACTCGTCCGTCTTCAACAACGCCATAGCTATGCTCCTGGGCAACGACATGAAC

*ScGAMYB* RXL10 ACAACCGACTCGTCCGTCTTCAACAACGCCATAGCTATGCTCCTGGGCAACGACATGAAC

*ScGAMYB* L35 ACAACCGACTCGTCCGTCTTCAACAACGCCATAGCTATGCTCCTGGGCAACGACATGAAC

*ScGAMYB* 541 ------------------------------------------------------------

*ScGAMYB* Ot1-3 ------------------------------------------------------------

*ScGAMYB* M12 ACGGAGTGCAAGCCTGTTTTCGGCGACGGTATCGTGTTTGATCATTCCTCGTGGAGCAAC

*ScGAMYB* DS2 ACGGAGTGCAAGCCTGTTTTCGGCGACGGTATCGTGTTTGATCATTCCTCGTGGAGCAAC

*ScGAMYB* RXL10 ACGGAGTGCAAGCCTGTTTTCGGCGACGGTATCGTGTTTGATCATTCCTCGTGGAGCAAC

*ScGAMYB* L35 ACGGAGTGCAAGCCTGTTTTCGGCGACGGTATCGTGTTTGATCATTCCTCGTGGAGCAAC

*ScGAMYB* 541 ------------------------------------------------------------

*ScGAMYB* Ot1-3 ------------------------------------------------------------

*ScGAMYB* M12 ATGCCGCATGCTTGTCAAATGTCGGAGGAGTTCAAATGA

*ScGAMYB* DS2 ATGCCGCATGCTTGTCAAATGTCGGAGGAGTTCAAATGA

*ScGAMYB* RXL10 ATGCCGCATGCTTGTCAAATGTCGGAGGAGTTCAAATGA

*ScGAMYB* L35 ATGCCGCATGCTTGTCAAATGTCGGAGGAGTTCAAATGA

*ScGAMYB* 541 ---------------------------------------

*ScGAMYB* Ot1-3 ---------------------------------------
