# Supplementary material for: The GAMYB gene in rye: sequence, polymorphisms, map location, allele-specific markers, and relationship with α-amylase activity
Source: BMC Genomics. 2020 Aug 24;21:578. doi: 10.1186/s12864-020-06991-3 (PMC7444254; doi:10.1186/s12864-020-06991-3)
Supplement: Supplementary file 3 — Additional file 3 Amino acid (aa) composition and polymorphisms of the protein translated from the ScGAMYB. Yellow backlight means differences in aa, pink backlight indicates changes that are important for the 2D protein structure and related to the effect of ScGAMYB on AMY. [file 12864_2020_6991_MOESM3_ESM.docx]

Additional file 3. Amino acid (aa) composition and polymorphisms of the protein translated from the *ScGAMYB*. Yellow backlight means differences in aa, pink backlight indicates changes that are important for the 2D protein structure and related to the effect of ScGAMYB on AMY.

1 10 20 30 40 50 60

| | | | | | |

*ScGAMYB* M12 MYRVKSESDCEMMHQEDQMDSPVGDDGSSGGSPHRGGGPPLKKGPWTSAEDAILVDYVKK

*ScGAMYB* DS2 MYRVKSESDCEMMHQEDQMDSPVGDDGSSGGSPHRGGGPPLKKGPWTSAEDAILVDYVKK

*ScGAMYB* RXL10 MYRVKSESDCEMMHQEDQMDSPVGDDGSSGGSPHRGGGPPLKKGPWTSAEDAILVDYVKK

*ScGAMYB* L35 MYRVKSESDCEMMHQEDQMDSPVGDDGSSGGSPHRGGGPPLKKGPWTSAEDAILVDYVKK

*ScGAMYB* Ot1-3 ------------------------------------------------------------

*ScGAMYB* 541 ------------------------------------------------------------

*ScGAMYB* M12 HGEGNWNAVQKNTGLFRCGKSCRLRWANHLRPNLKKGAFTPEEERLIIQLHSKMGNKWAR

*ScGAMYB* DS2 HGEGNWNAVQKNTGLNRCGKSCRLRWINYLRPDLKKGAFTPEEERLIIQLHSKMGNKWAR

*ScGAMYB* RXL10 HGEGNWNAVQKNTGLNRCGKSCRLRWINYLRPDLKKGAFTPEEERLIIQLHSKMGNKWAR

*ScGAMYB* L35 HGEGNWNAVQKNTGLNRCGKSCRLRWINYLRPDLKKGAFTPEEERLIIQLHSKMGNKWAR

*ScGAMYB* Ot1-3 ------------------------------------------------------------

*ScGAMYB* 541 ------------------------------------------------------------

*ScGAMYB* M12 MAAHLPGRTDNEIKNYWNTRIKRCQRAGLPVYPASVCNQSSNEDQQGSSDFNCGENLSSD

*ScGAMYB* DS2 MAAHLPGRTDNEIKNYWNTRIKRCQRAGLPVYPASVCNQSSNEDQQGSSDFNCGENLSSD

*ScGAMYB* RXL10 MAAHLPGRTDNEIKNYWNTRIKRCQRAGLPVYPASVCNQSSNEDQQGSSDFNCGENLSSD

*ScGAMYB* L35 MAAHLPGRTDNEIKNYWNTRIKRCQRAGLPVYPASVCNQSSNEDQQGSSDFNCGENLSSD

*ScGAMYB* Ot1-3 ------------------------------------------------------------

*ScGAMYB* 541 ------------------------------------------------------------

*ScGAMYB* M12 LLNGNGLYLPDFTCDNFIANSEALSYAPQLSAVSISSLLGQSFASKNCGFMDPVNQAGML

*ScGAMYB* DS2 LLNGNGLYLPDFTCDNFIANSEALSYAPQLSAVSISSLLGQSFASKNCGFMDQVNQAGML

*ScGAMYB* RXL10 LLNGNGLYLPDFTCDNFIANSEALSYAPQLSAVSISSLLGQSFASKNCGFMDPVNQAGML

*ScGAMYB* L35 LLNGNGLYLPDFTCDNFIANSEALSYAPQLSAVSISSLLGQSFASKNCGFMDPVNQAGML

*ScGAMYB* Ot1-3 -------------------------YAPQLSAVSISSLLGQSFASKNCGFMDQVNQAGML

*ScGAMYB* 541 -------------------------YAPQLSAVSISSLLGQSFASKNCGFMDQVNQAGML

*ScGAMYB* M12 KQSDPLLPGLSDTINGALSSVDQFSNDSEKLKQALGFDYLHEANSSSKIIAPFGGALTGS

*ScGAMYB* DS2 KQSDPLLPGLSDTINGALSSVDQFSNDSEKLKQALGFDYLHEANSSSKIIAPFGGALTGS

*ScGAMYB* RXL10 KQSDPLLPGLSDTINGALSSVDQFSNDSEKLKQALGFDYLHEANSSSKIIAPFGGALTGS

*ScGAMYB* L35 KQSDPLLPGLSDTINGALSSVDQFSNDSEKLKQALGFDYLHEANSSSKIIAPFGGALTGS

*ScGAMYB* Ot1-3 KQSDPLLPGLSDTINGALSSVDQFSNDSEKLKQALGFDYLHEANSSSKIIAPFGGALTGS

*ScGAMYB* 541 KQSDPLLPGLSDTINGALSSVDQFSNDSEKLKQALGFDYLHEANSSSKIIAPFGGALTGS

*ScGAMYB* M12 HAFLNGTFSTSRTINGPLKMELPSLQDTESDPNSWLKYTVAPAMQPTELVDPYLQSPTAT

*ScGAMYB* DS2 HAFLNGTFSTSRTISGPLKMELPSLQDTESDPNSWLKYTVAPAMQPTELVDPYLQSPTAT

*ScGAMYB* RXL10 HAFLNGTFSTSRTINGPLKMELPSLQDTESDPNSWLKYTVAPAMQPTELVDPYLQSPTAT

*ScGAMYB* L35 HAFLNGTFSTSRTINGPLKMELPSLQDTESDPNSWLKYTVAPAMQPTELVDPYLQSPTAT

*ScGAMYB* Ot1-3 HAFLNGTFSTSRTISGPLKMELPSLQDTESDPNSWLKYTVAPAMQPTELVDPYLQSPTAT

*ScGAMYB* 541 HAFLNGTFSTSRTISGPLKMELPSLQDTESDPNSWLKYTVAPAMQPTELVDPYLQSPTAT

*ScGAMYB* M12 PSVKSESASPRNSGLLEELLHEAQGLRSGKNQQLSVRSSSSSVSTPCDTTVVSPEFDLCQ

*ScGAMYB* DS2 PSVKSECASPRNSGLLEELLHEAQGLRSGKNQQLSVKSSSSSVSTPCDTTVVSPEFDLCQ

*ScGAMYB* RXL10 PSVKSESASPRNSGLLEELLHEAQGLRSGKNQQLSVRSSSSSVSTPCDTTVVSPEFDLCQ

*ScGAMYB* L35 PSVKSESASPRNSGLLEELLHEAQGLRSGKNQQLSVRSSSSSVSTPCDTTVVSPEFDLCQ

*ScGAMYB* Ot1-3 PSVKSESASPRNSGLLEELLHEAQGLRSGKNQQLSVRSSSSSVSTPCDTTVVSPEFDLCQ

*ScGAMYB* 541 PSVKSECASPRNSGLLEELLHEAQGLRSGKNQQLSVRSSSSSVSTPCDTTVVSPEFDLCQ

*ScGAMYB* M12 EYWEERLNEYAPFSGNSLTGSTAPVSTASPDVFQLSKISPAQSPSLGSGEQAMEPAYELG

*ScGAMYB* DS2 EYWEERLNEYAPFSGNSLTGSTAPVSTASPDVFQLSKISPAQSPSLGSGEQAMEPAYELG

*ScGAMYB* RXL10 EYWEERLNEYAPFSGNSLTGSTAPVSTASPDVFQLSKISPAQSPSLGSGEQAMEPAYELG

*ScGAMYB* L35 EYWEERLNEYAPFSGNSLTGSTAPVSTASPDVFQLSKISPAQSPSLGSGEQAMEPAYELG

*ScGAMYB* Ot1-3 EYWEERLNEYAPFSGNSLTGSTAPVSTASPDVFQLSKIPPA-------------------

*ScGAMYB* 541 EYWEERLNEYAPFSGNSLTGSTAPVSTASPDVFQLSKIPPA-------------------

*ScGAMYB* M12 AGDTSSHPENLRPDAFFSGNTTDSSVFNNAIAMLLGNDMNTECKPVFGDGIVFDHSSWSN

*ScGAMYB* DS2 AGDTSSHPENLRPDAFFSGNTTDSSVFNNAIAMLLGNDMNTECKPVFGDGIVFDHSSWSN

*ScGAMYB* RXL10 AGDTSSHPENLRPDAFFSGNTTDSSVFNNAIAMLLGNDMNTECKPVFGDGIVFDHSSWSN

*ScGAMYB* L35 AGDTSSHPENLRPDAFFSGNTTDSSVFNNAIAMLLGNDMNTECKPVFGDGIVFDHSSWSN

*ScGAMYB* Ot1-3 ------------------------------------------------------------

*ScGAMYB* 541 ------------------------------------------------------------

*ScGAMYB* M12 MPHACQMSEEFK

*ScGAMYB* DS2 MPHACQMSEEFK

*ScGAMYB* RXL10 MPHACQMSEEFK

*ScGAMYB* L35 MPHACQMSEEFK

*ScGAMYB* Ot1-3 ------------

*ScGAMYB* 541 ------------
